# Penalized likelihood and multi-objective spatial scans for the detection and inference of irregular clusters

**DOI:** 10.1186/1476-072X-9-55

**Published:** 2010-10-29

**Authors:** André LF Cançado, Anderson R Duarte, Luiz H Duczmal, Sabino J Ferreira, Carlos M Fonseca, Eliane CDM Gontijo

**Affiliations:** 1 Department of Statistics, Universidade de Brasília, Brasília/DF, Brazil; 2 Department of Mathematics, Universidade Federal de Ouro Preto, Ouro Preto/MG, Brazil; 3 Department of Statistics, Universidade Federal de Minas Gerais, Belo Horizonte/MG, Brazil; 4 Department of Electronic Engineering and Informatics, Universidade do Algarve, Portugal; 5 CEG-IST, Universidade Técnica de Lisboa, Portugal; 6 Department of Preventive and Social Medicine, Universidade Federal de Minas Gerais, Belo Horizonte/MG, Brazil

## Abstract

**Background:**

Irregularly shaped spatial clusters are difficult to delineate. A cluster found by an algorithm often spreads through large portions of the map, impacting its geographical meaning. Penalized likelihood methods for Kulldorff's spatial scan statistics have been used to control the excessive freedom of the shape of clusters. Penalty functions based on cluster geometry and non-connectivity have been proposed recently. Another approach involves the use of a multi-objective algorithm to maximize two objectives: the spatial scan statistics and the geometric penalty function.

**Results & Discussion:**

We present a novel scan statistic algorithm employing a function based on the graph topology to penalize the presence of under-populated disconnection nodes in candidate clusters, the *disconnection nodes cohesion function*. A disconnection node is defined as a region within a cluster, such that its removal disconnects the cluster. By applying this function, the most geographically meaningful clusters are sifted through the immense set of possible irregularly shaped candidate cluster solutions. To evaluate the statistical significance of solutions for multi-objective scans, a statistical approach based on the concept of attainment function is used. In this paper we compared different penalized likelihoods employing the geometric and non-connectivity regularity functions and the novel disconnection nodes cohesion function. We also build multi-objective scans using those three functions and compare them with the previous penalized likelihood scans. An application is presented using comprehensive state-wide data for Chagas' disease in puerperal women in Minas Gerais state, Brazil.

**Conclusions:**

We show that, compared to the other single-objective algorithms, multi-objective scans present better performance, regarding power, sensitivity and positive predicted value. The multi-objective non-connectivity scan is faster and better suited for the detection of moderately irregularly shaped clusters. The multi-objective cohesion scan is most effective for the detection of highly irregularly shaped clusters.

## Background

### Introduction

Spatial cluster detection and inference methods are important tools in geographical disease surveillance [1-8]. Irregularly shaped spatial clusters occur naturally in epidemiology and disease surveillance. The spatial scan statistic, defined as a likelihood ratio, is the usual measure of the strength of a cluster [9,10]. The circular scan [11], a particular case of the spatial scan statistic, is the most popular method for the detection and inference of disease clusters. Nevertheless, situations where spatial disease clusters do not have regular shape (e.g. non-circular or non-square shaped clusters) are fairly common. Clusters with arbitrary shape are found along traffic ways, plumes of air pollution, or geographical features such as rivers, shores and valleys. Many heuristics were developed recently to find arbitrarily shaped clusters [12-40] and are reviewed by Duczmal et al. [41].

The number of possible cluster candidates increases exponentially with the number of regions in a map. Even though all cluster solutions could be known, selecting the best cluster is an ill posed problem. The immense number of candidate solutions concurs to reduce the power of detection, because high likelihood ratio clusters can be constructed simply by adjoining elevated risk regions, forming very irregularly shaped clusters. Those clusters spread through large portions of the study area and do not bring useful information about the location of possible legitimate clusters, which are generally smaller and have somewhat lower likelihood ratio values. Thus there is motivation for using some kind of penalty function to avoid excessive freedom of clusters' shape. Other measures for the strength of a cluster must be taken into consideration, such as geometric [21,22,24,28] or non-connectivity (graph-based) [38] regularity. All cluster detection methods for irregular shaped clusters uses some means to restrict the extent of the cluster, even though it may be very weak, such as a simple connectivity requirement.

The geometric regularity penalty function acts as a low-pass filter, reducing the value of clusters which are very different from a circle, which is the most compact geometric shape. Without penalization, a very irregularly shaped cluster almost always wins against a compact, more regularly shaped cluster; with penalization, there is a chance that the more compact cluster wins, depending on the amount of contrast it delineates against the whole study area. The non-connectivity regularity function penalizes more a cluster whose associated adjacency graph has fewer edges, given its number of nodes. In other words, the most penalized clusters are those whose graphs are trees, which are loosely connected by definition. Although both geometric and topological regularity functions do provide sensible measures of cluster strength, it could be argued that sometimes we really would not want to penalize every kind of irregular shape or non-connectivity; many interesting and geographically meaningful clusters are really irregular, and there is no reason to avoid them.

The concept of *disconnection node *was briefly discussed elsewhere [21]. A disconnection node is a region within a cluster which disconnects it when removed, splitting the cluster into two or more connected pieces. In the present paper we argue that the presence of under-populated disconnection nodes impacts the power of detection of clusters. It happens because it is more difficult to aggregate loosely connected pieces which are glued through small population regions. A novel regularity function, the *disconnection nodes cohesion function*, is defined in order to measure the strength of a cluster, based on the presence or absence of under-populated disconnection nodes.

A multi-objective genetic algorithm was developed elsewhere to identify irregularly shaped clusters [18,24]. That method conducts a search aiming to maximize two objectives, namely the scan statistic and the regularity of shape (using the geometric compactness concept). The presented solution is a set of non-dominated solutions, consisting of those clusters which were found not to be worse then any other known cluster in both objectives simultaneously. The multi-objective approach has an advantage over penalized likelihood methods: all potential clusters are considered for comparison without altering their ranking due to penalty modifications. Thus the ranking decision is executed only after all the candidates are evaluated. Penalized methods otherwise decide beforehand the amount of applied penalty, being prone to distortions in the process of choosing the most likely cluster. Multi-objective methods eliminate all but a small set of potential *non-dominated solutions*, the candidate clusters which are not worse than any other candidate in both objectives simultaneously.

The significance evaluation is conducted in parallel for all the clusters in the non-dominated solutions set using a Monte Carlo simulation, breaking the tie among them and determining the best cluster solution. In this paper we present the statistical theory of attainment functions to compute the clusters' significance. The use of the attainment function allows us to extend in a natural way the meaning of the *p*-value to the bi-objective space, while preserving the dependence between points within the same non-dominated set, for all non-dominated sets obtained by the Monte Carlo simulation. This contrasts with the previous approaches, in which the sets of candidates solutions were "dissolved" into independent points, leading to a loss of information about the distribution of non-dominated sets among the objective space under the null hypothesis [18,24].

In the present paper we compare three multi-objective scan methods using the geometric compactness, the non-connectivity and the disconnection node functions as one objective and the spatial scan statistic as the second objective. Those methods are compared with the corresponding single-objective likelihood penalized methods. Their power to detect irregularly shaped spatial clusters, sensitivity and positive predicted value are studied through numerical simulations. The C language codes are available from the corresponding author.

We summarize the concepts of Kulldorff's spatial scan statistic and review the single-objective genetic algorithm, the geometric regularity function, the non-connectivity graph-based regularity function and the multi-objective genetic algorithm. We introduce the novel disconnection nodes cohesion function and the attainment functions to compute the clusters' significance. The single-objective and multi-objective scans are evaluated through numerical simulations. We apply those methods to find spatial clusters of Chagas' disease in puerperal women in Minas Gerais state, Brazil.

### The Spatial Scan Statistic

In this section we review the spatial scan statistic [9], its implementation through a genetic algorithm [22], the geometric [21] and the non-connectivity [38] regularity functions, and the multi-objective spatial scan statistic [24].

A study area map *A *is divided into *M *regions, with total population *N *and *C *total cases. A graph denoted by *G_A _*is associated with the study area *A *with *M *nodes representing the regions and edges linking nodes corresponding to adjacent regions. A zone is any collection of connected regions. Under the null hypothesis there are no clusters in the map, and the number of cases in each region is Poisson distributed proportionally to its population. For each zone *z*, the number of observed cases is *c_z _*and the expected number of cases under null hypothesis is *μ_z _*= *C*(*n_z_/N*), where *n_z _*is the population in the zone *z*. The relative risk of *z *is *I*(*z*) = *c_z_/μ_z _*and the relative risk of the complement of *z *is *O*(*z*) = (*C *- *c_z_*)/(*C *- *μ_z_*). Defining *L*(*z*) as the likelihood function under the alternative hypothesis and *L*_0 _as the likelihood function under the null hypothesis, it can be shown (see [9] for details) that the logarithm of the likelihood ratio for the Poisson model is given by:

(1) $$\begin{array}{c} LLR(z)=\log\left(\frac{L(z)}{L_{0}}\right)\\ LLR(z)=\{\begin{array}{ll} c_{z}\text{log}(I(z))+(C-c_{z})\text{log}(O(z)) & \text{if}\;I(z)>1\\ 0 & \text{otherwise} \end{array} \end{array}$$

It is maximized over the chosen set *Z *of potential zones *z*, identifying the zone that constitutes the *most likely cluster*. For instance, when the set *Z *contain the zones defined by circular windows of different radii and centers, max_*z*∈*Z *_*LLR*(*z*) is the circular scan statistic [11]; when *Z *contain all the zones defined by elliptical windows of different sizes, centers, elongations and orientations, max_*z*∈*Z *_*LLR*(*z*) is the elliptic scan statistic [28]. When *Z *is the set of all connected zones, the evaluation of every zone of *Z *is not feasible in practice for large maps (although [42] claims that maps up to about 100 nodes are attainable using the GraphScan algorithm), and many heuristics have appeared recently to compute approximate values for max_*z*∈*Z *_*LLR*(*z*) [41]. Those heuristics (often called irregularly shaped spatial scan statistics) employ stochastic algorithms to explore the set of configurations *Z *or even evaluate a restricted subset of *Z*. The statistical significance of the most likely cluster of observed cases is computed through a Monte Carlo simulation, according to Dwass [43]. Under null hypothesis, simulated cases are distributed over the study area and the scan statistic is computed for the most likely cluster. This procedure is repeated thousands of times, and the distribution of the obtained values is compared with the *LLR *of the most likely cluster of observed cases, producing its p-value.

### The geometric penalty function

Most irregularly shaped spatial cluster detection algorithms frequently end up with a cluster solution that is merely a collection of the high incidence regions, linked together forming a "tree-shaped" zone spread out through the map; the associated sub-graph resembles a tree, possibly except for some few additional edges. In general it is hard to give a geographical meaning for this kind of cluster, because this kind of solution does not add any new information with regard to its special location in the map. One easy way to avoid that problem is simply to set an upper bound to the maximum number of cells within a zone. This approach is only effective when cluster size is rather small (i.e., for detecting clusters occupying roughly up to 10% of the regions of the map). For larger upper bounds in size, the increased geometric freedom favors the occurrence of very irregularly shaped tree-like clusters, thus impacting the power of detection. The geometric compactness penalty for irregularly shaped clusters was presented by Duczmal et al. [21], penalizing the zones in the map that are highly irregularly shaped. For this purpose the geometric compactness *K*(*z*) of a zone *z *is defined as the area of *z *divided by the area of the circle with the same perimeter as the convex hull of *z*. Compactness is dependent on the shape of the object, but not on its size. Compactness also penalizes a shape that has small area compared to the area of its convex hull. The circle is the most compact shape (*K*(*z*) = 1). The compactness penalyzed scan statistic is defined as *max*_*z*∈*Z *_*LLR*(*z*)*:K*(*z*). A user defined exponent *a *is attached to *K*(*z*) in order to control its strength; the resulting scan statistic is then *max*_*z*∈*Z *_*LLR(z):K(z)*^*a*^. Larger values of *a *increase the effect of the penalty, allowing the presence of more compact clusters only. Similarly, lower values of *a *allow for more freedom in shape. The idea of using a penalty function for spatial cluster detection, based on shape irregularity, was first used for ellipses [28], although a different formula was employed.

### The non-connectivity penalty function

Yiannakoulias et al. [38] proposed a greedy algorithm to explore the space *Z *of all possible zones *z*. A new non-connectivity penalty function was based on the ratio of the number of edges *e*(*z*) to the number of vertices *v*(*z*) in the candidate cluster *z*. The non-connectivity penalty was employed as a multiplier to *LLR*(*z*), analogously to the geometric compactness penalty. In the same way, a user defined exponent *a *is attached to the non-connectivity penalty to control its strength. Although the non-connectivity penalty is in many ways similar to the geometric compactness penalty, it has an important difference: it does not rely on the geometric shape of the candidate cluster, which could be an interesting advantage when searching for real clusters which are highly irregularly shaped, but present good connectivity properties.

### Single-Objective Genetic Algorithms

Conley et al. [16] proposed a genetic algorithm to explore a configuration space of multiple agglomerations of ellipses for point data sets. The method employed a strategy to "clean-up" the best configuration found in order to geometrically simplify the cluster. Sahajpal et al. [36] used a genetic algorithm to find clusters in point data sets, shaped as the intersections of circles with different sizes and centers.

A genetic algorithm was developed [22] for spatial cluster detection and inference using the scan statistic as the test statistic in a map divided into regions. The algorithm aims to maximize an objective function (in this case the Kulldorff's spatial scan statistic expression(1)), by modifying an initial set of individuals, or genetic population, for a number of generations. The *crossover *and *mutation *operators increase the variability of the population. The *selection *operator chooses the individuals that will remain in the next generation, maintaining a fixed genetic population size. The crossover operator creates new offspring individuals, or zones, mixing the features of two randomly chosen parents *A *and *B*, which are themselves zones from the previous generation. Several children are thus produced, which are intermediate zones between the two extreme zones *A *and *B*. The mutation operator introduces random perturbations in the features of one individual zone, (either adding or removing one random region) thus increasing the variability of the population. The selection operator ranks the zones according to the value of the objective function, namely the spatial scan statistic. We expect to find individuals with increasingly higher values of the objective function as the algorithm advance through the generations. The graph-related operations are minimized by means of a fast offspring generation and evaluation of Kulldorff's spatial scan statistic. A geometric compactness penalty function is employed to avoid excessive irregularity of the cluster geometric shape. This algorithm is an order of magnitude faster and exhibits less variance compared to other algorithms [22], like the simulated annealing scan [19], and is more flexible than the elliptic scan. It has about the same power of detection as the simulated annealing scan for mildly irregular clusters and is superior for the very irregular ones.

### Multi-objective Genetic Algorithms

Duczmal et al. in [24] have proposed an approach to the geographic delineation of irregularly shaped disease clusters, treating it as a multi-objective optimization problem. Most current spatial scan software usually displays only one of the many possible cluster solutions with different shapes. In the multi-objective approach, the best cluster solutions are found by maximizing two competing objectives simultaneously: regularity of shape *K*(*z*), and scan statistic value *LLR*(*z*). The compactness *K*(*z*) is no longer used as a penalty correction, but as a new objective function instead. That approach simplifies the problem and allows a stronger grasp of the question of finding the "best" cluster solution. The amount of penalization applied is not an issue in multi-objective methods. Genetic algorithms are well suited for treating multi-objective optimization problems, evolving a set of tentative solutions towards the optimal solutions in parallel [44,45].

The pairs (*LLR_i_, K_i_*), representing the logarithm of the scan statistic value and compactness computed for each individual *i *(connected set of regions in the map) in the genetic population, are plotted in the Cartesian plane. The selection operator uses the concept of dominance: a point is called *dominated *if it is worse than another point in at least one objective, while not being better than that point in any other objective [46]. The *non-dominated set *consists of all solutions which are not dominated by any other solution.

The construction of the initial population and the crossover and mutation operators are identical to those used in the single objective genetic algorithm (see [22] for a detailed description of those operators). At the beginning of each generation, we compute the current generation list, which consists of the set of parent individuals augmented several times with the addition of newly produced offspring through the crossover operator. The next generation list, initially empty, stores the individuals that will survive for the next generation. We compute the set *P*_0 _of non-dominated solutions of the current generation list, which is transferred to the initially empty next generation list; the same set *P*_0 _is also removed from the current generation list. A new set *P*_1 _of non-dominated solutions of the remaining individuals is computed from the actual current generation list, and the procedure is repeated until the new generation list has grown to contain *M *individuals, where *M *is the number of regions of the original map and corresponds to the population size that will be held constant along the generations. After a number of steps, say *l*, the set *P_l _*will eventually not be totally added to the next generation list, because this would cause the list to contain more than *M *individuals. In such cases, the individuals of *P_l _*are transferred randomly, one by one, until the next generation list contains exactly *M *individuals. This procedure is known as *non-dominated sorting *[47].

In the context of irregularly shaped clusters, the first of the competing objectives (regularity of shape) could not be considered appropriate if it was the only objective of the search. If so, we would inevitably obtain a circularly shaped, but possibly meaningless, solution. Conversely, consider the complementary situation, when the maximization of the likelihood ratio, irrespective of shape, is the only objective: as we have seen in the introduction, this would also produce solutions which are not useful from a geographic perspective. The maximization of shape regularity only makes sense when coupled with the maximization of likelihood ratio, as developed in the multi-objective methodology. Isolated, neither objective is sufficient to guide the search for the most likely clusters, when we have the freedom to choose among clusters of arbitrary shape. A rather regularly shaped cluster usually has many neighborhood connections with its adjacent regions, compared to the number of component regions within the cluster, due to the fact that its compactness is high. Otherwise, an irregularly shaped cluster is probably "tree-like" in the sense that the number of connections with adjacent regions is small compared to the number of component regions. In a situation where two clusters have the same *LLR*, and one is more regularly shaped than the other, the former is preferred: the compactness of a cluster is generally related to the strength with which its component regions connect to each other. In this regard, compactness is considered as a measure of stability of the cluster, as a solid geographic entity: we probably can remove a few regions from a regularly shaped cluster without breaking it apart, but a similar operation may not be possible for a highly irregularly shaped cluster.

## Methods

In this section we define the novel disconnection nodes cohesion function and introduce the attainment surface concept to compute the clusters' statistical significance. The multi-objective scans are compared through numerical simulations and an application for Chagas' disease clusters is presented.

### The Disconnection Nodes Cohesion Function

Consider a study area map *A *associated with its non-directed graph *G_A_*, and a connected zone *z *with the corresponding connected sub-graph *G *= (*V, E*) of *G_A_*. The nodes in set *V *correspond to the regions of *z *and each non-directed edge (*i, j*) in set *E *occurs whenever the regions *i *and *j *share a common boundary. A node *x *∈ *V *is called a *disconnection node *of *G *if the sub-graph obtained from *G *with the nodes set *V - *{*x*} is not connected. Let *D *= {*x*_1, _*..., x_d_*} ⊂ *V *be the set of all the disconnection nodes of *G*. For each *x_i _*∈ *D*, let *pop*(*x_i_*) be the population of the region associated with node *x_i_*. Let $\mu_{x_{i}}$ be the expected number of cases of the region corresponding to node *x_i _*under the null hypothesis, which is proportional to *pop*(*x_i_*). The sub-graph with the nodes set *V - D*, obtained from *G*, consists of the *L remaining connected subgraphs *${\hat{z}}_{1},\;.\;.\;.\;,\;{\hat{z}}_{L}$, where 2 ≤ *L *≤ |V| - *d*. Let *pop*(${\hat{z}}_{j}$) be the population of the *remaining connected zone *associated with ${\hat{z}}_{j}$. The *L *connected parts ${\hat{z}}_{1},\;.\;.\;.\;,\;{\hat{z}}_{L}$ are ranked in decreasing order according to their populations, as ${\hat{z}}_{\left(1\right)},\;.\;.\;.\;,\;{\hat{z}}_{\left(L\right)}$.

The cohesion function of the sub-graph *G *is now defined as:

(2) $$c(G)=\{\begin{array}{ll} \left(\prod_{i=1}^{d}(1-e^{-\mu_{x_{i}}})\right)\prod_{i=1}^{L}\frac{pop\left({\hat{z}}_{\left(i\right)}\right)}{\sum_{j=i}^{L}pop\left({\hat{z}}_{\left(j\right)}\right)} & \text{if}\;D\;\text{is}\;\text{not empty}\\ 1 & \text{otherwise} \end{array}$$

If each region has non-zero population, then 0 *< c*(*G*) ≤ 1.

If we assume that the number of cases $c_{x_{i}}$ in each disconnecting node *x_i _*∈ *D *is a Poisson random variable with mean $\mu_{x_{i}}$, then the factor $1-e^{–\mu_{x_{i}}}$ is equal to *P *($c_{x_{i}}$*>*0), the probability of the number of the cases being greater than zero. It is important to note that we are only aggregating the individual contributions of each disconnecting node of the candidate cluster, without assuming independence with respect to the product over the disconnecting nodes factors $1-e^{–\mu_{x_{i}}}$. Thus the first term in the cohesion formula penalizes those zones which have low values of $\mu_{x_{i}}$.

The second term penalizes homogeneous population distribution among the *L *connected subgraphs ${\hat{z}}_{1},\;.\;.\;.\;,\;{\hat{z}}_{L}$: it is understood that the presence of disconnecting nodes which break the cluster apart more evenly (regarding their populations) strongly impacts its cohesion. Otherwise, breaking the cluster more heterogeneously, i.e., leaving large parts of it intact while breaking away only the remaining lowly populated connected parts is considered less damaging to its cohesion.

**Figure 1:**
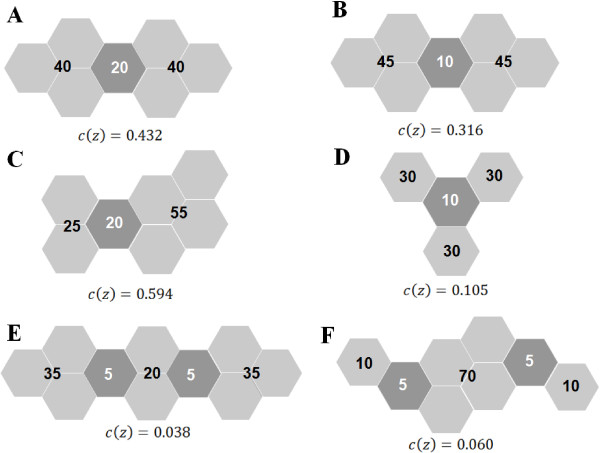
**Disconnection nodes cohesion function evaluation for several clusters**.

Figure 1 presents six clusters *A*-*F *where the regions are represented by hexagons. The disconnecting nodes are indicated by dark gray hexagons. Each cluster consists of one or two disconnecting nodes and two or three remaining connected zones(represented by connected sets of light gray hexagons). Each remaining connected zone carries a number representing its population. The value of the cohesion function *c*(*z*) is displayed below each cluster.

Consider that the study area has a total of 100 cases and population 1, 000. Each cluster has population 100. Then the cohesion value for cluster *E*, for instance, is computed as:

$$c(z)={\left(1-\exp\;(-0.1\times 5)\right)}^{2}\left(\frac{35}{35+35+20}\right)\;\;\left(\frac{35}{35+20}\right)\;\;\left(\frac{20}{20}\right)=0.038$$

Clusters *A *and *B *differ in the population size of their disconnection nodes. Cluster *A *has larger cohesion and is considered more structurally stable because its two remaining zones are linked by a disconnection node with larger population.

Clusters *A *and *C *differ in the population heterogeneity of their remaining zones. When removed from cluster *C*, the disconnection node leaves a relatively large remaining connected zone of population 55 intact. Cluster *C *has larger cohesion and is considered more structurally stable because its two remaining zones have very different populations, compared with the two evenly distributed remaining zones of cluster *A*. Cluster *D *illustrates the effect of splitting the cluster into more than two remaining connected zones. Compared to cluster *B*, cluster *D *has very low cohesion due to the fact that it is split into three equally populated remaining zones after the removal of the disconnection node.

The removal of the two disconnection nodes in clusters *E *and *F *produces three remaining connected zones in each cluster. The three remaining connected zones of cluster *E *are more homogeneously distributed than the corresponding ones of cluster *F*. Consequently, cohesion for cluster *F *is higher, due to the fact that the central remaining connected zone of cluster *F *has relatively higher population 70.

The algorithm always verifies if a cluster consisting of only one single region is itself a good solution, as the addition of new regions to this possible cluster also would lead to rapid decrease in LLR value. That node alone constitutes by itself a potential solution without including any additional disconnecting nodes.

The non-connectivity penalty function penalizes weakly connected clusters, measuring the proportion of the number of nodes over the number of edges. We pursue this notion further in our cohesion penalty function, indicating which nodes contribute more to the resulting "weakness" of the cluster. We are interested in penalizing clusters where the only connection between areas consists of a low populated area. If the algorithm detects a cluster for which the removal of a low populated area does not break it, it means that other "paths" of connection between different areas are still present, and thus the cluster could be considered as a solid geographic entity; otherwise it should be better regarded as a weak union of two or more secondary clusters. By the other hand, highly populated areas which disconnect the cluster when removed do not contribute so incisively to weaken the cluster; this happens because the path through this highly populated area, linking other nodes of the cluster, is considered important enough to impart strength to the structure.

Summarizing, the presence of a low population disconnecting node is an obstacle to the formation of good solutions, especially when its removal breaks the candidate cluster such that the largest remaining piece is considerably smaller than the original cluster.

When used as a penalty factor, *c*(*G*) is incorporated in the expression (1) for the test statistic as a multiplier for the log likelihood ratio, meaning that the penalization is strong when the cohesion function assumes lower values (there is no penalization at all when *c*(*G*) = 1).

**Figure 2:**
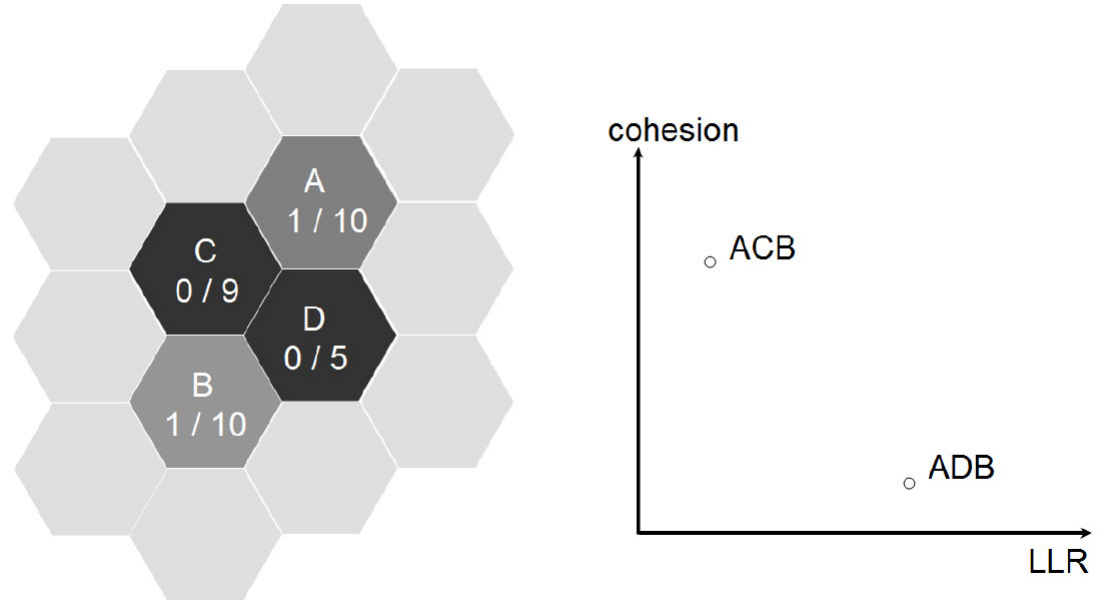
**LLR and disconnection nodes cohesion for the clusters *ACB *and *ADB***.

When *c*(*G*) is used as the second objective in the multi-objective version of the genetic algorithm both the test statistic *LLR*(*z*) (1) and the disconnection node cohesion function are maximized. In the example in Figure 2, two clusters *ACB *and *ADB*, are evaluated. The number of cases *X *and population *Y *are represented as "X/Y" for each region. Cluster *ACB *has disconnection node *C *and population 29, and cluster *ADB *has disconnection node *D *and population 25 (both have 2 cases). Cluster *ACB *has lower *LLR *than cluster *ADB*, but cluster *ACB *has greater cohesion than cluster *ADB*, due to the larger population of the disconnection node *C*, and neither dominates the other (see the graph to the right in Figure 2).

### Computing the clusters' statistical significance

Consider a bi-objective maximization problem with objective functions *f*_1 _and *f*_2_. Let E = {*x_j_*, *j *= 1, ..., *Q*} be the set of all solutions evaluated in an optimization run, and define its image $I=\{Y_{j}=(f_{1}(x_{j}),f_{2}(x_{j})),j=1,...,Q\}$, contained in the objective space ℝ^2^. As mentioned in the Background section, the solution *x_j _*is called *non-dominated *if *x_j _*is not dominated by any other solution in E. Let $\{x_{j}^{*},j=1,...,q\}\subset E$ be the set of non-dominated solutions of E. The subset $Y=\{Y_{j}^{*}=(f_{1}(x_{j}^{*}),f_{2}(x_{j}^{*})),j=1,...,q\}\subset I$ is defined as the *outcome *of a single run of a bi-objective algorithm.

**Figure 3:**
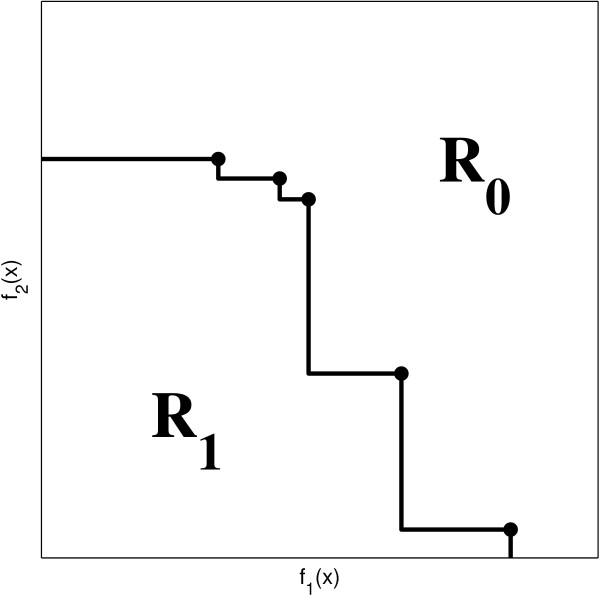
**The attainment surface splits the objective space in two regions**.

**Figure 4:**
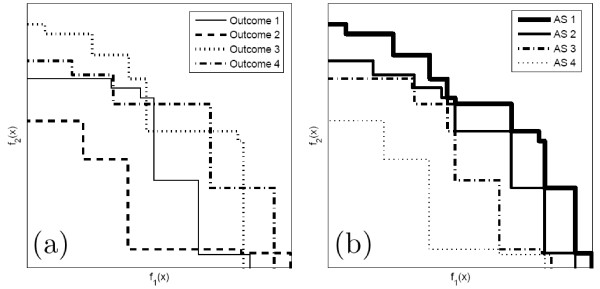
**(a) Outcomes obtained by multiple runs of a biobjective algorithm and (b) the corresponding estimated attainment surfaces**.

We can associate with Y a boundary which splits the objective space in two regions *R*_1 _and *R*_0_: *R*_1 _is the region consisting of points dominated by, or equal to, at least one point in Y and *R*_0 _consists of the points that are not dominated by any of the points in Y (see Figure 3). When the solution *x *is dominated by at least one solution of a given outcome Y, we say that *x *is *attained *by Y. In Figure 3, any solution located in the region *R*_1 _is attained by Y. Now consider *n *runs of the algorithm. As each run produces distinct outcomes we will obtain multiple boundaries, as in Figure 4(a).

Points lying in the upper right of the figure were not attained in any of the runs. Points that lie in the lower left were attained in all the runs. And points lying between different outcomes were attained in some runs but not in others. So we can split the space in *n *+ 1 types of regions according to the frequency at which these regions are attained. The boundaries of these regions are called *attainment surfaces *[48](see Figure 4(b)). These frequencies are used to estimate the probability of attaining a point in the objective space, when a large number of runs is executed.

The attainment function [48,49] evaluated at some point *Y *in the objective space can be estimated by the outcome sets $Y_{1},\;.\;.\;.\;,\;Y_{n}$ obtained through *n *independent runs of the algorithm, as

$$A_{n}(Y)=\frac{1}{n}\sum_{i=1}^{n}\;I\;(Y_{i}\underline{⊳}Y)$$

where the symbol "$\underline{⊳}$" means that $Y_{i}$ attains *Y *and **I **is the indicator function having value 1 if $Y_{i}\underline{⊳}Y$, and value zero otherwise.

**Figure 5:**
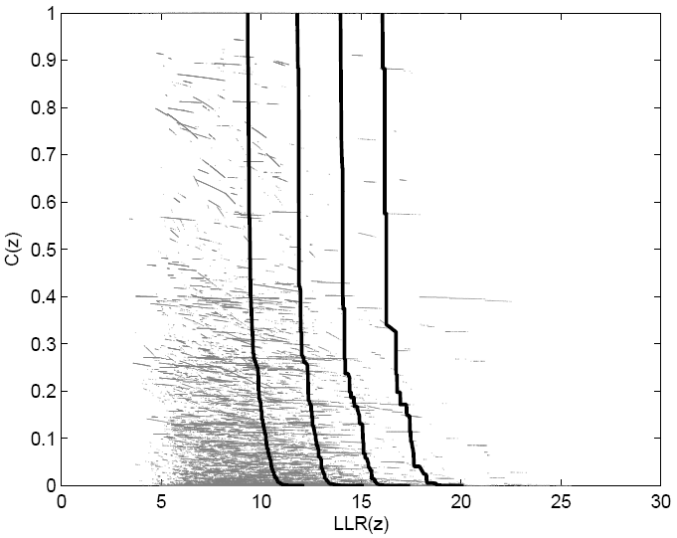
**The 0.316, 0.1, 0.032 and 0.01 p-value isoline curves for the null hypothesis Monte Carlo simulation, using 1,000 non-dominated sets**.

In the specific problem of the present paper we are interested in estimating the p-value of non-dominated candidate cluster solutions represented by points in the (*LLR*, *Mes*) objective space, where *Mes *is the desired measure, such as compactness, non-connectivity or cohesion, discussed in the previous sections. Formally, we define *A*(*Y *) as the $\underset{n\to \infty }{\lim}A_{n}(Y)$ when it exists. Now, given 0 *< p *≤ 1 the *p-value isoline *is defined as the inverse image *A*^-1^(*p*). For sufficiently smooth conditions, *A*^-1^(*p*) is an 1-dimensional surface dividing the objective space into two regions *R*_0 _and *R*_1_, such that if *Y *∈ *R*_1 _then *A*(*Y*) *> p*, and if *Y *∈ *R*_0 _then *A*(*Y*) ≤ *p*. In practice, given *n *outcome sets $Y_{1},\;.\;.\;.\;,\;Y_{n}$ we can construct approximations of the p-value isolines for every *p *= *i*/(*n *+ 1),*i *= 1, ..., *n *through the estimated attained function *A_n_*(*Y*). The example of Figure 5 displays some p-value isolines resulting from *n *= 1000 outcome sets under the null hypothesis. The outcome points are displayed in gray.

When a stochastic algorithm is used, only part of the potential set of solutions is evaluated, and there is no guarantee that the optimal non-dominated solutions are found. This of course could lead to a biased estimation of the significance, producing underestimated p-values. Thus the computed p-values are in fact lower bounds for the theoretical p-values.

## Results and Discussion

### Numerical Evaluations

In this section we compare numerically the disconnection nodes cohesion scan (DN), the geometric compactness scan (GC), the non-connectivity scan (NC) and the no-penalty genetic scan (NP). We set the value of the exponent *a *= 1 (see *The geometric penalty function *and *The non-connectivity penalty function *subsections) for all single-objective scans.

We also compare the corresponding multi-objective scans: the multi-objective disconnection nodes cohesion scan (MDN), the multi-objective geometric compactness scan (MGC) and the multi-objective non-connectivity scan (MNC). We evaluate their power of detection, sensitivity and positive predicted value (PPV).

**Figure 6:**
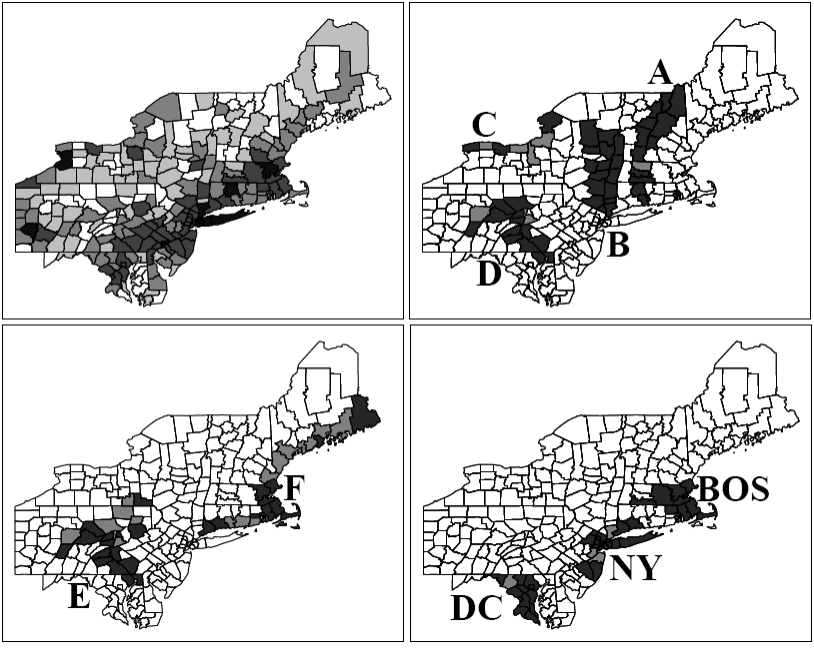
**Simulated data clusters for the 245 counties northeastern U.S map**. In the upper left map, shades indicate counties populations. On the remaining three maps, the clusters A-F, DC, NY and BOS were used in the power evaluations. Lighter shades indicate disconnection nodes.

A benchmark dataset for real data population for breast cancer of the Northeastern US is used [21]. This benchmark consists of 245 counties in 10 states and the District of Columbia, with a total population at risk of 29,535,210 women. The upper left map of Figure 6 display the county population quantiles by shades of gray. Nine simulated irregularly shaped clusters, *A*-*F *, *NY *, *BOS *and *D.C*., are displayed in the remaining three maps of (Figure 6). These clusters were chosen for the purpose of testing the limits of the algorithms for some very irregular cluster shapes. Clusters *NY *, *BOS *and *D.C*. are located in highly populated areas, contrasting with the remaining clusters, which are located in rural or mixed areas defined roughly by geographic features such as rivers or shores (see [21]). The lighter shade regions indicate disconnection nodes inside the clusters. All clusters have at least one disconnection node, except *B *and *BOS *which have *c*(*z*) = 1. Clusters *F *, *C *and *E *have the lowest disconnection nodes cohesion.

From now on, those clusters will be denoted *real *clusters, in contrast to the *detected *clusters found by the algorithms. For each simulation of data under these nine alternative hypotheses, 600 cases are distributed randomly according to a Poisson model using a single cluster; we set a relative risk equal to one for every cell outside the real cluster, and greater than one and identical in each cell within the cluster. The relative risks for each cluster are defined such that if the exact location of the real cluster was known in advance, the power to detect it should be 0.999 [50].

Given an alternative hypothesis model, 5, 000 runs of the multi-objective algorithm produce the correspondent non-dominated sets, which are joined and compared to the 0.05 p-value isoline, obtained under null hypothesis through 10, 000 Monte Carlo replications. The proportion of non-dominated sets which have at least one point located to the right of the 0.05-value isoline is an estimate of the *power *of the algorithm for that particular alternative hypothesis model.

Additionally, we perform the three corresponding null hypotheses simulations of 10, 000 runs. The measures of sensitivity and PPV also serve to evaluate the quality of the cluster detection process. These measures can be defined in the terms of the population size. We define sensitivity and PPV as:

$$\begin{array}{l} Sensitivity\;=\;\;\frac{Pop(\text{Detected}\;\text{C}1\text{uster}\;\cap \;\text{Rea}1\;\text{Cluster})}{Pop(\text{Rea}1\;\text{C}1\text{uster})}\\ \;PPV=\;\;\frac{Pop(\text{Detected}\;\text{C}1\text{uster}\;\cap \text{Rea}1\;\text{Cluster})}{Pop(\text{Detected}\;\text{C}1\text{uster})} \end{array}$$

For the single-objective scans, the three measures of power, sensitivity an PPV were computed for the most likely cluster in each replication. For the multi-objective scans, they were computed based on the smallest p-value cluster in their non-dominated set for all significant sets. Tables 1, 2 and 3 presents the average power, sensitivity and PPV for 5, 000 replications of each of the nine alternative hypotheses clusters A-F, NYC, BOS and D.C. for all the seven scans. For each cluster the highest measurement among the single-objective and the multi-objective scans were presented in bold type.

**Table 1:** Power comparisons for the single-objective algorithms and multi-objective algorithms.

cluster	NP	GC	NC	DN	MGC	MNC	MDN
A	0.838	0.822	0.881	0.839	0.950	0.942	0.946
B	0.882	0.843	0.926	0.898	0.954	0.969	0.958
C	0.827	0.814	0.826	0.667	0.933	0.915	0.932
D	0.896	0.840	0.922	0.877	0.962	0.965	0.968
E	0.874	0.778	0.885	0.822	0.947	0.946	0.950
F	0.629	0.433	0.585	0.510	0.746	0.743	0.795
NY	0.759	0.747	0.819	0.868	0.888	0.909	0.914
BOS	0.792	0.834	0.864	0.892	0.918	0.928	0.937
D.C.	0.803	0.903	0.877	0.901	0.955	0.936	0.936

**Table 2:** Positive predicted value comparisons for the single-objective algorithms and multi-objective algorithms.

cluster	NP	GC	NC	DN	MGC	MNC	MDN
A	0.624	0.578	0.665	0.619	0.803	0.711	0.666
B	0.699	0.691	0.786	0.765	0.781	0.821	0.755
C	0.625	0.344	0.659	0.582	0.716	0.734	0.676
D	0.696	0.616	0.771	0.734	0.751	0.803	0.743
E	0.719	0.633	0.762	0.704	0.760	0.785	0.740
F	0.664	0.314	0.650	0.565	0.710	0.729	0.714
NY	0.898	0.621	0.929	0.941	0.918	0.942	0.928
BOS	0.781	0.389	0.827	0.861	0.891	0.854	0.825
D.C.	0.788	0.518	0.865	0.887	0.931	0.882	0.847

**Table 3:** Sensitivity comparisons for the single-objective algorithms and multi-objective algorithms.

cluster	NP	GC	NC	DN	MGC	MNC	MDN
A	0.796	0.551	0.792	0.767	0.732	0.748	0.828
B	0.707	0.598	0.784	0.743	0.702	0.767	0.763
C	0.851	0.360	0.796	0.607	0.735	0.749	0.854
D	0.668	0.506	0.713	0.668	0.629	0.656	0.703
E	0.534	0.414	0.544	0.508	0.514	0.507	0.551
F	0.583	0.170	0.523	0.430	0.519	0.524	0.632
NY	0.580	0.364	0.650	0.643	0.572	0.638	0.629
BOS	0.747	0.295	0.806	0.841	0.692	0.743	0.810
D.C.	0.725	0.426	0.791	0.802	0.748	0.756	0.788

### Power

The three multi-objective scans were superior in terms of power of detection. In fact, Table 1 shows that, for all nine clusters, the lowest power of detection among the three multi-objective scans was higher than the power of all the four single-objective scans. The MDN scan was the best multi-objective scan on five clusters, and the second best on the remaining four clusters. The NC scan was the best performing single-objective scan on four clusters and the second best on other four. The variation in power for the multi-objective scans is very small (about 2%), contrasting with the large variation found in the single-objective scans. For the very irregularly shaped cluster F, there is a considerable gain in power of detection when using multi-objective scans, compared to the single objective scans.

### PPV

Regarding PPV, Table 2 shows that, for all nine clusters, at least one of the multi-objective scans have obtained a better result than the best result among the four single-objective scans. Also, on average, the multi-objective scans performed better than the single-objective scans. The MNC scan was the best performing multi-objective scan on six clusters and the second best on the remaining three. The NC scan was the best among the single-objective scans on five clusters and the second best on the remaining four. For the very irregularly shaped cluster F, all the multi-objective scans have better results compared with every one of the four single-objective scans.

### Sensitivity

Table 3 shows that, on average, the multi-objective scans performed better than the single-objective scans in terms of sensitivity. Among the multi-objective scans, the MDN scan received the best evaluation on seven clusters, and was the second best on the remaining two clusters. The NC scan was the best performing single-objective scan on four clusters and the second best on the remaining five. For the very irregularly shaped cluster F, the MDN scan was the only penalty function based scan to surpass the performance of the NP single-objective scan.

### Chagas' Disease clusters

**Figure 7:**
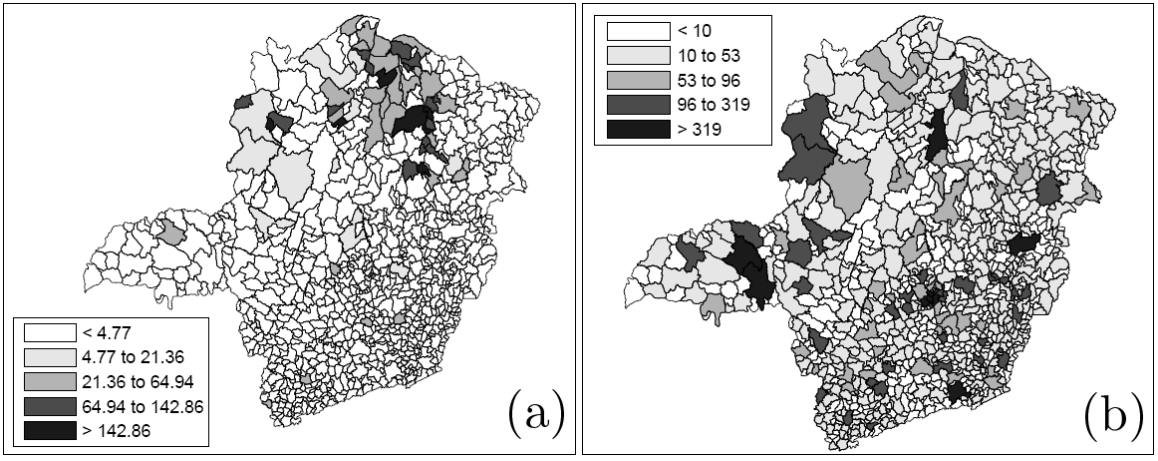
**(a) Maps of rates (per one thousand individuals) of Chagas' disease and (b) populations at risk in the state of Minas Gerais, Brazil**.

Chagas' disease is caused by the parasite *Trypanosoma cruzi*. It is transmitted to animals and people by blood-sucking insect vectors (triatomine bugs), which are found only in the Americas. The disease is found chiefly in poor rural areas of Latin America. An individual can be infected if the parasite present in the bug's feces enter the body through mucous membranes, the bite wound itself or others breaks in the skin. Other ways of infection include: consumption of uncooked food contaminated with feces from infected bugs; congenital transmission (from a infected pregnant woman to her baby); blood transfusion and organ transplantation. In recent years, due to better control of the triatomine bug infestation, the congenital transmission became one of the main transmission mechanism of the Chagas infection. In this work we study the occurrence of Chagas' disease in puerperal women in the state of Minas Gerais, located in Brazil's southeast. The population at risk consists of women that gave birth to babies in the period of July to September, 2006. The new-born babies were blood tested to detect the presence of the Chagas disease antigen, with coverage above 96%. A positive test means that the mother is infected. These tests were conducted through the project PETN-MG (Minas Gerais State Program for New-Born Screening) coordinated by the research group NUPAD-MEDICINA/UFMG from Federal University of Minas Gerais Medical School http://www.nupad.medicina.ufmg.br in collaboration with Minas Gerais State Health Secretary. The state is divided into 853 municipalities with a total population at risk of 24, 969 women. After a comprehensive screening to eliminate false positives a total number of 113 cases were obtained. In Figure 7 the incidence map (cases per 1000 women) for each municipality is shown on the left, and the quantile population map is shown on the right. Most municipalities have zero cases in the period. To detect clusters, we apply the circular scan, the non-penalty single-objective genetic scan and our three multi-objective scans described in the previous sections. We have used a maximum cluster size of 40 for all methods, based on the satisfactory results obtained by the circular scan for this size.

**Figure 8:**
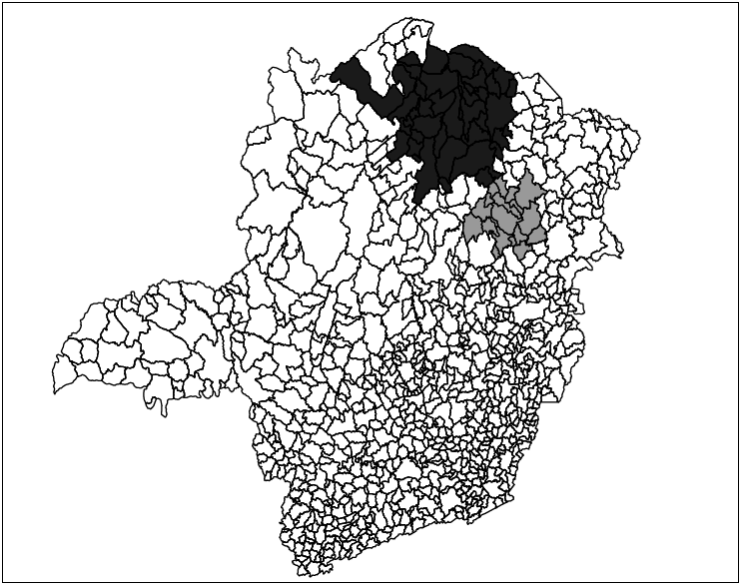
**Primary (darker shade) and secondary (lighter shade) Chagas' disease clusters detected by the circular scan**.

**Figure 9:**
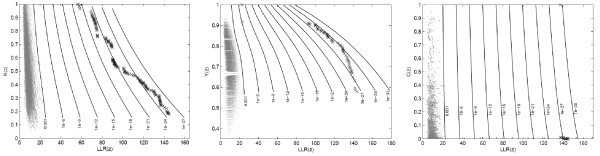
**Isoline curves and observed clusters (crosses) found by MGC, MNC and MDN scans, from left to right, respectively**. Isolines were obtained by extrapolation of the 1,000 non-dominated sets, indicated by gray points.

**Figure 10:**
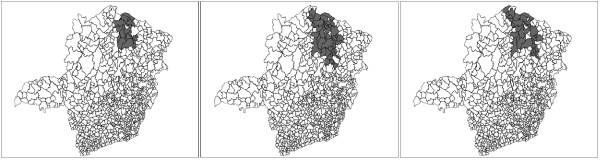
**Most likely clusters found by GC, NC and DN algorithms, from left to right, respectively**.

**Figure 11:**
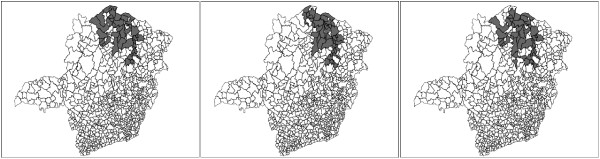
**Most likely clusters found by MGC, MNC and MDN algorithms, from left to right, respectively**.

The primary and secondary circular scan clusters are shown to the right in Figure 8 and Table 4. The three graphs of Figure 9 display the complete set of non-dominated solutions for the MDN, MGC and MNC scans respectively. The '×' symbols represent the clusters in the non-dominated solution sets obtained by the three scans consist of respectively 150, 63 and 12 clusters. The gray points at the left part of each graph represents 1000 non-dominated solution sets simulated under the null hypothesis. For each scan the most likely cluster was selected among the clusters of the non-dominated solution set of the observed cases map according to its smallest estimated p-value. The p-value isolines represent constant p-values for the clusters found under the null hypothesis, ranging from 10^-3 ^to 10^-27 ^or less at the rightmost line. Those p-values are estimated through extrapolation from the 1000 null hypothesis non-dominated solutions sets using the attainment function method. Employing the Gumbel semi-parametric model [24,51], we have assumed that the p-values decrease according to the logarithm of the LLR. Of course there is a large amount of uncertainty about the precise location of those very small p-values isolines, but the relevant features here are the overall isolines' slopes [24]. The point representing the most likely cluster is distinguished among the non-dominated solution set according to these slopes. For the three GC, NC and DN single-objective scans, and the three MGC, MNC and MDN multi-objective scans, the most likely clusters are presented in the maps of Figures 10 and 11 respectively. Table 4 displays the number of regions, LLR, the respective measures values (for compactness, non-connectivity and cohesion), population, number of cases, rate and estimated p-value for the most likely clusters found. The p-values shown in the table are conservative estimates based only on counting for the 1000 Monte Carlo simulations, but they are in fact much smaller (less than 10^-24^), as can be inferred from Figure 9. The most likely cluster found by the single-objective GC scan is considerably smaller than the cluster found by the MGC scan. Similarly, the cluster found by the DN scan is also smaller than the cluster found by the MDN scan. Otherwise, the cluster found by the single-objective NC scan is almost identical to the cluster found by the MNC scan.

**Table 4:** Chagas' disease clusters of the non-dominated set of Figures 8, 10, 11.

Scan	*n*(*z*)	*LLR*	measures	pop	cases	rate × 1000	p-value
circular prim.	40	87.5	-	1,444	57	39.47	*<*0.001
circular sec.	18	13.6	-	453	13	28.70	*<*0.001
GC	20	69.1	*k*(*z*) = 0.947	491	35	71.28	*<*0.001
NC	32	116.2	*y*(*z*) = 0.816	1,330	65	48.87	*<*0.001
DN	40	126.3	*c*(*z*) = 1.000	1,460	70	47.95	*<*0.001
MGC	40	134.9	*k*(*z*) = 0.319	1,634	75	45.90	*<*0.001
MNC	40	128.3	*y*(*z*) = 0.798	1,487	71	47.75	*<*0.001
MDN	40	137.4	*c*(*z*) = 1.000	1,732	77	44.46	*<*0.001

**Figure 12:**
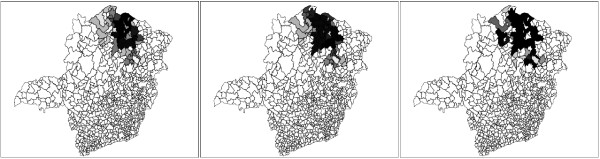
**Gray scale maps for the observed non dominated solutions obtained by MGC, MNC and MDN algorithms, from left to right, respectively**.

Each of the three remaining maps of Figure 12 display simultaneously all clusters in the respective MGC, MNC and MDN scans' non-dominated solution sets. A gray color coding scheme was used to indicate the proportional number of times each region of the map was present in the non-dominated solution set, from black (the region was present in all clusters) to white (the region was not present in any cluster). This gray scale representation helps the practitioner to distinguish those regions which appear in almost all clusters, thus being part of the "core" of the cluster. Note that this core usually does not match exactly the most likely cluster, and constitute an additional tool for identifying the most prevalent regions of the cluster.

## Conclusions

We compared penalized likelihood and multi-objective methods for the detection and inference of spatial disease clusters employing Kulldorff's spatial scan statistics. The penalized likelihood methods maximize the product of a regularity function by the likelihood ratio scan statistic over the set of potential clusters, employing a genetic algorithm. Regularity functions evaluate a potential cluster, in terms of its geometric shape or topological graph structure, and are used to control the excessive freedom in the shape of the clusters. The novel disconnection node cohesion function was introduced in this work and compared with two previous regularity functions, the geometric compactness and the non-connectivity functions. The cohesion function uses the graph topology to penalize the presence of under-populated disconnection nodes in candidate clusters, the *disconnection nodes cohesion function*. A disconnection node is defined as a region within a cluster, such that its removal disconnects the cluster. By applying this function, the most geographically meaningful clusters are sifted through the immense set of possible irregularly shaped candidate cluster solutions.

The disconnection cohesion penalty function discourages the presence of a node with zero cases, unless this node is well connected with the remaining of the cluster. It is important to note that we are primarily concerned with low population disconnecting nodes in the set of potential solutions. Otherwise, a node with high population and small number of cases would lead to a rapid decrease in the LLR value for the potential cluster under study, thus producing an unattractive solution by the algorithm. The disconnection nodes cohesion regularity function penalizes inconsistent clusters, on the same time allowing the presence of the geographically interesting irregularly shaped ones. It penalizes irregularly shaped clusters selectively: the irregularity is allowed only to the extent that it does not impact the stability of the cluster, or its sensitivity to the removal of disconnection nodes.

The algorithm tests only single disconnected nodes. Consider the situation where a single node does not break the cluster, but an adjacent group of two or more nodes break it. One should then generalize the algorithm to take into account all those possible adjacent groups instead of the single nodes, as we have defined. Of course, the number of possible situations should be prohibitively large. We have conducted a numerical experiment, employing single and double nodes to check if this modification should alter the performance of the algorithm. No significant improvements were observed, although the running time increased ten-fold. For this reason, we decided to use only single disconnected nodes.

Multi-objective scans maximize two objectives: the spatial scan statistics and a chosen regularity function. All three regularity functions, namely, compactness, non-connectivity and cohesion were used to build the corresponding three multi-objective scans. Their power of detection, sensitivity and positive predicted value were compared with the corresponding penalized likelihood scans, the non-penalty genetic algorithm scan and the usual circular scan. The variation in power of detection for the three multi-objective scans is very small. In all situations they were superior to all the single-objective scans. On average, the multi-objective scans have higher positive predicted value than the single-objective scans, with the multi-objective non-connectivity scan showing the best results. The multi-objective scans also have higher average sensitivity than the single-objective scans. The multi-objective cohesion scan displayed the highest sensitivity. For very irregularly shaped clusters, the multi-objective cohesion scan performed consistently better than the other scans with regard to power, positive predicted value and sensitivity.

The run time for 1000 Monte Carlo replications for the Northeast US breast cancer cluster analysis, using an Intel core i7 3.33*GHz *desktop, took about 21 minutes for the MGC scan, 27 minutes for the MDN scan and only 4.5 minutes for the MNC scan. The corresponding run times for the single objective scans are almost the same. With present desktop computers, cluster analyses for maps containing 1000 nodes are relatively easy, and even larger maps analyses are doable.

Each multi-objective scan is not significantly slower than the corresponding single-objective scan. The non-connectivity scan is about six times faster than the other scans. In summary, our simulations suggest that the three multi-objective scans have better performance than the single-objective scans. We recommend the multi-objective non-connectivity scan as a relatively fast algorithm for the detection of moderately irregularly shaped clusters, and the multi-objective cohesion scan for the detection of highly irregularly shaped clusters.

Genetic algorithms in general do require careful parameters calibration and their application to the detection of spatial clusters may not appear very user friendly, e.g. compared to the circular scan. Despite of that, our experience shows that the optimal parameter selection (mutation rate, survival rules for the next generation, etc.) in the genetic algorithm based scans is very robust and do not change much for different geographical maps.

We applied the attainment function methodology to extend the meaning of the *p*-value to the bi-objective space in a natural way. This approach may be compared with [18,24]. In these previous works, the sets of candidates solutions were "dissolved" into independent points, leading to a loss of information about the distribution of non-dominated sets among the objective space under the null hypothesis. The attainment function preserves the dependence between points within the same non-dominated set, for all non-dominated sets obtained by the Monte Carlo simulation, producing a comparatively more robust definition for cluster significance in multi-objective scans.

An application was presented for Chagas' disease incidence in puerperal women in Brazil. This study case is particularly difficult to analyze, due to the parsity of cases and the presence of many regions with zero cases, which could potentially produce a large uncertainty in the delineation of clusters. It should be noted that all three multi-objective scans' most likely clusters are not very dissimilar. The cohesion scan cluster is slightly more irregularly shaped than the other two clusters.

We plan to combine the effects of the compactness, non-connectivity and the cohesion functions in a future work.

## Competing interests

The authors declare that they have no competing interests.

## Authors' contributions

ALFC, ARD, LHD, SJF and CMF devised the methods used in the study, wrote the necessary computer programs, conducted the simulations and data analysis and wrote the manuscript. ECDMG provided the Chagas' disease data and assisted in the writing of the manuscript. All authors read and approved the final version of the manuscript.
